# Retraction Note: Long non-coding RNA linc00645 promotes TGF-β-induced epithelial–mesenchymal transition by regulating miR-205-3p-ZEB1 axis in glioma

**DOI:** 10.1038/s41419-024-06648-z

**Published:** 2024-04-17

**Authors:** Chenlong Li, Hongshan Zheng, Weiliang Hou, Hongbo Bao, Jinsheng Xiong, Wanli Che, Yifei Gu, Haiming Sun, Peng Liang

**Affiliations:** 1 Department of Neurosurgery, Harbin Medical University Cancer Hospital, Harbin, Heilongjiang 150001 PR China; 2 Laboratory of Medical Genetics, Harbin Medical University, Harbin, Heilongjiang 150001 PR China; 3 Key Laboratory of Medical Genetics (Harbin Medical University), Heilongjiang Higher Education Institutions, Harbin, Heilongjiang 150001 PR China

Retraction to: *Cell Death and Disease* 10.1038/s41419-019-1948-8, published online 26 September 2019

The Editors-in-Chief have retracted this article after the authors alerted them to problems with several of the figures. Further investigation by the Publisher has found the following concerns:

- Several image overlaps between Figure 2l and Figure 6E
- Image overlap between Figure 6A, sh-NC (35 days, first image in the second row) with Figure 6A, sh-NC (15 days, first image in the bottom row) of [1]
- Image overlap between Figure 6E, T89G (second and fourh image in the bottom row) with Figure 2D, U251 (both images in the bottom row) of [1]
- Image overlap in Figure 6F, U251, specifically between the seventh and eighth images in the second row

In addition, the authors have provided an ethics approval document which is dated after the start of glioblastoma and normal brain tissue collection from patients undergoing tumor resection. The Editors-in-Chief therefore no longer have confidence in the results and conclusions presented.

Chenlong Li agrees to this retraction. Peng Liang, Haiming Sun, Hongbo Bao, Wanli Che, Yifei Gu, Weiliang Hou, Jinsheng Xiong, and Hongshan Zheng have not responded to any correspondence from the editor/publisher about this retraction.
